# A New Font, Specifically Designed for Peripheral Vision, Improves Peripheral Letter and Word Recognition, but Not Eye-Mediated Reading Performance

**DOI:** 10.1371/journal.pone.0152506

**Published:** 2016-04-13

**Authors:** Jean-Baptiste Bernard, Carlos Aguilar, Eric Castet

**Affiliations:** 1 Aix-Marseille Université, Marseille, France; 2 Laboratoire de Psychologie Cognitive (UMR 7920), Fédération de Recherche 3C, CNRS, Marseille, France; University of Leicester, UNITED KINGDOM

## Abstract

Reading speed is dramatically reduced when readers cannot use their central vision. This is because low visual acuity and crowding negatively impact letter recognition in the periphery. In this study, we designed a new font (referred to as the Eido font) in order to reduce inter-letter similarity and consequently to increase peripheral letter recognition performance. We tested this font by running five experiments that compared the Eido font with the standard Courier font. Letter spacing and x-height were identical for the two monospaced fonts. Six normally-sighted subjects used exclusively their peripheral vision to run two aloud reading tasks (with eye movements), a letter recognition task (without eye movements), a word recognition task (without eye movements) and a lexical decision task. Results show that reading speed was not significantly different between the Eido and the Courier font when subjects had to read single sentences with a round simulated gaze-contingent central scotoma (10° diameter). In contrast, Eido significantly decreased perceptual errors in peripheral crowded letter recognition (-30% errors on average for letters briefly presented at 6° eccentricity) and in peripheral word recognition (-32% errors on average for words briefly presented at 6° eccentricity).

## Introduction

*"Faciliter et améliorer la lecture et l'écriture*, *c'est améliorer les communications entre les hommes; […] J'estime que celui qui parviendrait à débarrasser l'humanité des confusions*, *qui*, *dans les écritures rapides*, *s'établissent entre les u et les n*, *n'aurait pas à regretter sa peine*, *même si sa vie entière avait été consacrée à la conquête de ce progrès si minime en apparence*.*"*

*"Facilitating and improving reading and writing leads to improved communication between people; […] I believe that the one who would succeed in removing confusions*, *such as the one that exists between "u" and "n" in writing*, *would not regret this effort even if his/her whole life would have been devoted to such an apparently small endeavor*.*"*

*Emile Javal from his book*: *Physiologie de la lecture et de l'écriture*, *1905*.

Visual crowding is a perceptual limitation that impairs the recognition of flanked objects and increases with visual eccentricity [1–3]. It is considered as the main sensory factor limiting word recognition and reading [4] because it impairs the identification of groups of letters, the necessary primary step of word identification [5]. Based on this consideration, we would expect reduction in letter crowding to improve peripheral letter and word recognition, and consequently reading performance. The reason is that readers rely significantly on their parafoveal vision when they process information during reading [6]. This advantage should be even larger for readers who rely exclusively on their peripheral vision such as patients with central field loss (notably Age-Related Macular Degeneration patients). However, several experiments that reduced letter crowding have shown a null or modest effect on reading speed improvement. Chung et al added extra-spaces between letters to reduce letter crowding, without any significant improvement for central and peripheral reading speed (measured with a Rapid Serial Visual Presentation *or RSVP* paradigm) in normally-sighted readers [7] and patients with central field loss [8]. Chung and Mansfield [9] mixed the polarity of adjacent letters within words, which led to a reduction in letter crowding but did not improve peripheral RSVP reading speed. One explanation for these results could be that adding extra-spacing or changing contrast polarity did not sufficiently improve crowded letter recognition [9,10] in order to improve reading speed, in part because adding extra-spacing also reduces the visual acuity of letters by shifting them further into the periphery [10,11]. Indeed, when letter crowding was largely reduced through perceptual learning [12–14], reading speed performance measured with a RSVP paradigm was significantly improved (more than 60% in reading speed in [12]). Adding extra spaces between words (increasing interline and/or inter-word spacings) was also shown to have a modest effect on RSVP and page-mode reading speed performance [15–17].

The errors that occur during letter crowding theoretically limit reading speed [4]. They can usually be classified in two categories: mislocation errors and confusion errors [18–20]. Letter mislocation errors correspond to cases in which observers confuse the target with one of the flankers [21–23]. These errors represent between 20% and 35% of letter identification errors for a traditional alphabet of 26 letters in different studies [18,20]. Letter confusion errors correspond to the confusion of the target letter with a letter that is not present among the flankers. Interestingly, confusion errors occurring during letter crowding are not uniformly distributed and certain pairs of letters are more frequently confused. For instance, a confusion matrix from a recent study [18] showed that 50% of the confusion errors that occurred during a crowded letter recognition task with Courier letters were caused by only 36 pair confusions (5% of all the possible pair confusions). Letter confusion errors also occur for single letters presented very briefly [24], in noise [25], in the periphery [26], at low contrast [27], for a print-size close to the acuity [28] or even for tactile letter recognition tasks [29]. Models that predict single letter confusion errors exist based on optical and neuronal limitations [30–32], but models that predict crowded letter confusion errors are missing, mainly because attentional and neuronal mechanisms of crowding are not clearly defined [3]. However, some similarity exists between single and crowded letter confusions [33,34]. Different studies (with single or crowded letter recognition) showed that the pairs of letters that are frequently confused have evident physical similarities [26,35–38]. For instance, Bouma [26] used the 26 letters from the Courier font to define seven different groups of letters based on their physical similarities: (1) Inner parts and rectangular envelope (a, s, z, x), (2) Round envelope (e, o, c), (3) Vertical outer parts (n, m, u), (4) Oblique outer parts (r, v, w), (5) Ascending extensions (d, h, k, b), (6) Slenderness (t, i, l, f) and (7) Descending parts (g, p, j, y, q). When letters were presented close to their acuity threshold in central and peripheral vision, he observed that confusions occurred almost exclusively between letters from the same group. He pointed out that cues for recognition are limited in impaired vision, increasing confusions between letters sharing similar features. The specificity of these confusions suggests that increasing distinctiveness among similar letters should reduce confusion errors that occur during peripheral single and crowded letter recognition. This was observed with simple letter physical modifications in peripheral single letter recognition [39].

In this study, we tested the hypothesis that reducing inter-letter physical similarity could improve crowded letter recognition by creating a new font that we called the Eido font. We also minimized Eido letter complexity, another factor that is hypothesized to increase letter crowding [18,40,41]. Finally, because we wanted to minimize the learning time for the Eido letters, our letter templates were built with usual characteristics of lowercase and uppercase letters present in traditional fonts such as Arial, Courier or Times. As word recognition and reading are preceded by letter recognition [5], we predicted that a significant improvement in peripheral letter recognition performance would improve peripheral word recognition and reading speed performance. To anticipate our results, we found that Eido improved static letter recognition and word recognition performance as well as it improved word recognition processing, but not eye-mediated sentence reading. This suggests that improving letter and word recognition performance is not sufficient to counter limitations that occur when eye movements are involved in peripheral sentence reading.

## Material and Methods

### Subjects

Twelve young subjects with normal or corrected-to-normal vision and aged from 22 to 37 participated in this study. The research followed the tenets of the Declaration of Helsinki and was approved by the Ethical Committee for Protection of Human Subjects at the Aix-Marseille Université. Written informed consent was obtained from each observer after the nature and purpose of the experiment had been explained. Six subjects participated in Experiments 1, 2 and 3 and 5. Six other subjects participated in Experiment 4. Three subjects who participated in experiments 1, 2, 3 and 5 also participated in Experiment 6 (control experiment).

### Apparatus

Stimuli were displayed on a 21-inch CRT color monitor (ViewSonic P227f, refresh rate = 120 Hz, resolution = 1152 x 854 pixels) driven by a PC computer running custom software developed in Python with the Psychopy library [40]. Observers sat in a comfortable chair with their eyes at a distance of 40 cm from the monitor in a dimly lit room (screen visual angle: 50.8° x 37.7°). They viewed the screen with their dominant eye while wearing a patch over the contralateral eye and with their head maintained against a forehead rest. An eyetracker (Eyelink 1000 Tower Mount distributed by SR Research Ltd., Mississauga, Ont., Canada) was connected to our system. It was used to simulate a central scotoma in Experiment 1 and 5, and to control the gaze fixation of observers in Experiment 2, 3 and 4. Letters, words and other stimuli were displayed in black (luminance: 0.3 cd/m^2^) on a light gray background (luminance: 60 cd/m^2^).

### Artificial scotoma

A gaze-contingent ten-degree round central scotoma was simulated in Experiment 1 and 5 to measure peripheral reading speed for each of our subjects by using an Eyelink eye tracker with procedure described in [15,42]. The scotoma was a gray mask (same luminance of the background). The pupil of the dominant eye was tracked at a frequency of 1,000 Hz and used to display the scotoma centered on the gaze position with a delay less than 10ms [15,42]. A 9-point gaze calibration followed by a 9-point gaze validation was performed before each experimental block (length of a block: 20 sentence trials in Experiment 1 and 15 sentence trials in Experiment 5). Each trial was triggered by the observer who pressed a button while he/she was fixating a central dot. This was used to perform an offset correction (called “drift correction” in the Eyelink terminology) at the beginning of each trial.

### Letter trigrams, words and reading material

Sentences used in Experiment 1 were carefully controlled and already used in one of our previous experiments [15]. They were extracted from Alexandre Dumas' books obtained from the Project Gutenberg book database (www.gutenberg.org). The sentences were selected to have lengths, including spaces and commas, between 40 and 60 characters, and to contain words from the 20,000 most frequent words in written French, according to the word-frequency table obtained from the Lexique 3 database (http://www.lexique.org). Only sentences that contained no punctuation other than a period or comma were used. Accents and apostrophes, which are very common in French, were accepted characters. The period at the end of each sentence was not displayed. With these constraints, inspired by the rules used in the MNREAD test [43], a total of 2261 sentences were generated.

Letter trigrams used in Experiment 2 (and 6) were trigrams that can be found in French words (i.e. they were not strings of random letters). The probability of presenting a trigram in our crowded letter recognition experiment depended on its frequency in French words obtained using word frequencies from the Lexique 3 database (http://www.lexique.org).

Four hundred French words were used in Experiment 3. They were French word lemmas made of 4 to 10 characters, with a range of lexical frequencies from 5 to 454 occurrences per million (frequencies from the Lexique 3 database).

Three hundred French words and three hundred French pseudowords were used in Experiment 4. We prepared these sets by extracting six hundred words that were single-morpheme French word lemmas made of 4 to 7 characters, with a range of orthographic neighborhood from 4 to 9 and a range of lexical frequencies from 10 to 454 occurrences per million. From this set, three hundred French words were directly extracted, and the other three hundred words were used to build three hundred pseudowords based on the following principle: For each word, one interior letter was replaced with another letter to create a string of letters with the same orthographic and phonologic characteristics of a real word, but that was not part of the French dictionary [44].

Sentences used in Experiment 5 were extracted from one short book from Prosper Mérimée: *L'enlèvement de la redoute*. These sentences were read one after the other in the right order so that each reader could follow the story of the book. On average, sentences were made of 15.89±8.15 words or 91.41±46.16 characters (average±sd). Note that reading speeds for subjects with central scotoma usually cannot be measured with paragraphs made of a large numbers of characters because of the large print-size that is usually used. Based on these constraints, single-sentence reading speeds were measured in experiments 1 and 5.

### Design of the Eido font

**Fig 1:**
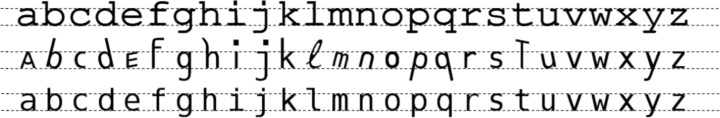
Description of the Courier (top-row), Eido (middle-row) and DejaVu (bottom-row) fonts used in our experiments. Note that the letter x-height and center-to-center spacing were the same for the different fonts.

The lowercase version of the Eido font used in this study was designed with the free software Fontforge (www.fontforge.org). The 26 letter templates of the Eido font were inspired by the letter templates of the DejaVu font (http://dejavu-fonts.org/wiki/Main_Page), a font with traditional letter templates similar to Courier, but a smaller complexity (a smaller ink area compared to the Courier font for the same x-height). The Eido font letters are shown in Fig 1 and have been designed following three main principles: (1) Reducing physical similarities between letters, (2) Reducing letter visual complexity and (3) Designing letters that observers are familiar with. To design the Eido font, we carefully examined frequent pair confusions (based on confusion matrices) occurring in previous studies [18,24,26,27,34,36] and changed/created physical features to reduce the physical similarities between confusable letters. In particular, our goal was to break the similarity groups defined by Bouma [26]. Some of these groups were based on letter envelopes, defined by Bouma as the smallest enclosing polygon without indentations, which appears directly linked to the low spatial frequency information of a letter. Thus, we modified the physical appearance of letters within the rectangular envelope group (letters a, s, z and x) by replacing the letter 'a' with its uppercase traditional template (letters 'z' and 'x' were not changed because of their low occurrences). We did the same with the round envelope group (letters e, o and c), by replacing the letter 'e' with its uppercase template. The horizontal bars of the letter 'e' were also slightly tilted. The strokewidth of the letter ‘o’ was increased by 20% and the gap of the letter ‘c’ was extended. Concerning the vertical outer parts group (letters n, m and u), we tilted these three letters by 15°. Confusions being more frequent between letters 'n' and 'u', we tilted 'm' and 'n' with a clockwise orientation compared to 'u' that was tilted with an anti-clockwise orientation. Concerning ascending extensions (d, h, k, b), we tilted by 15° the letters 'b' (clockwise orientation) and ‘d’ (anti-clockwise orientation) and the top of the letter 'h' was curved. For the slenderness group (letters t, i, l and f), we raised up and tilted the horizontal bar of the letter ‘t’, the dot on the letters ‘i' was enlarged and slightly moved away. The letter ‘l’, source of frequent confusions, was defined as its handwriting shape. Concerning the descending part group (letters g, p, j, y and q), we tilted the letters 'p' and 'q' similarly to the letters 'b' and 'd', and the dot on the letter 'j' was enlarged and slightly moved away identically to the letter 'i'. The letters 'k', 'v', 'w', 'x', 'y' and 'z' were not changed, given their rare occurrences.

Ascender and descender parts of the Eido letters have been vertically enlarged compared to usual fonts like Courier or Times to increase the inter-letter discriminability. Eido ascender line size was defined as 0.8x the x-height and Eido descender line size as 0.75x the x-height (Courier ascender line size is 0.4x the x-height and Courier descender line size is 0.4x the x-height).

### Comparison between the Eido, Courier and DejaVu fonts

In experiments 1, 2, 3, 4 and 5, we compared the Eido font with the Courier font, a traditional monospaced font, and in Experiment 6, we compared the DejaVu font with the Courier font. These three fonts are shown in Fig 1. For a stringent comparison between the three fonts, we were extremely careful in matching two influential parameters in crowded letter recognition and reading performance: (1) the letter x-height that corresponds to the letter height for letters that are not ascender or descender letters [45], and (2) the center-to center spacing that corresponds to the distance between each letter center [2]. Because letter-stroke width can have an effect on reading speed [46], we also slightly adjusted the Courier letter-stroke width to have the same stroke width for both fonts (about 0.15x the x-height). The low-complexity design of the Eido font causes a difference in the edge-to-edge spacings between the Eido (0.31*x-height, average edge-to-edge spacing for the 'abcdefghijklmnopqrstuvwxyz' letter string) and the Courier fonts (0.12*x-height), see Fig 1). Because of this, Experiment 6 was run with the DejaVu font, a font with usual letter shapes (very similar to the Courier font) but with an edge-to-edge spacing similar to the Eido font (the Eido font was originally derived from the DejaVu font). Interline spacing was the same for the Eido and Courier fonts during the sentence reading task (there was no reading task using the DejaVu font).

### Experimental protocol

We ran five experiments to compare the Eido and Courier fonts for (1) MNREAD-like eye-mediated sentence reading speed, (2) Crowded letter recognition rate, (3) Word recognition rate, (4) Lexical decision reaction time and (5) Eye-mediated reading speed with sentences from a novel. This latter experiment was always run after Experiments 1, 2 and 3 to test possible training effects. A sixth experiment comparing crowded letter recognition performance of the Courier and DejaVu fonts (fonts with similar edge-to-edge spacing) was run to ensure that a difference in edge-to-edge spacing was not the cause of the results observed in experiments 2, 3 and 4 with the Eido and Courier fonts. Results from Experiment 1 were used to set the letter print size used in experiments 2, 3, 4 and 5. Each subject ran a short reading practice (20 sentences with random print sizes for each font) with the simulated 10° round central scotoma before starting the Experiment 1. We used the lower visual field in our static-eye experiments 2, 3, 4 and 5 because: (1) The lower visual field is strongly suggested to be the most efficient location of the visual field to recognize words, and (2) The lower visual field is the peripheral locus that is mostly used by patients during word recognition and reading [47].

#### (1) Experiment 1: Sentence reading with simulated scotoma

**Fig 2:**
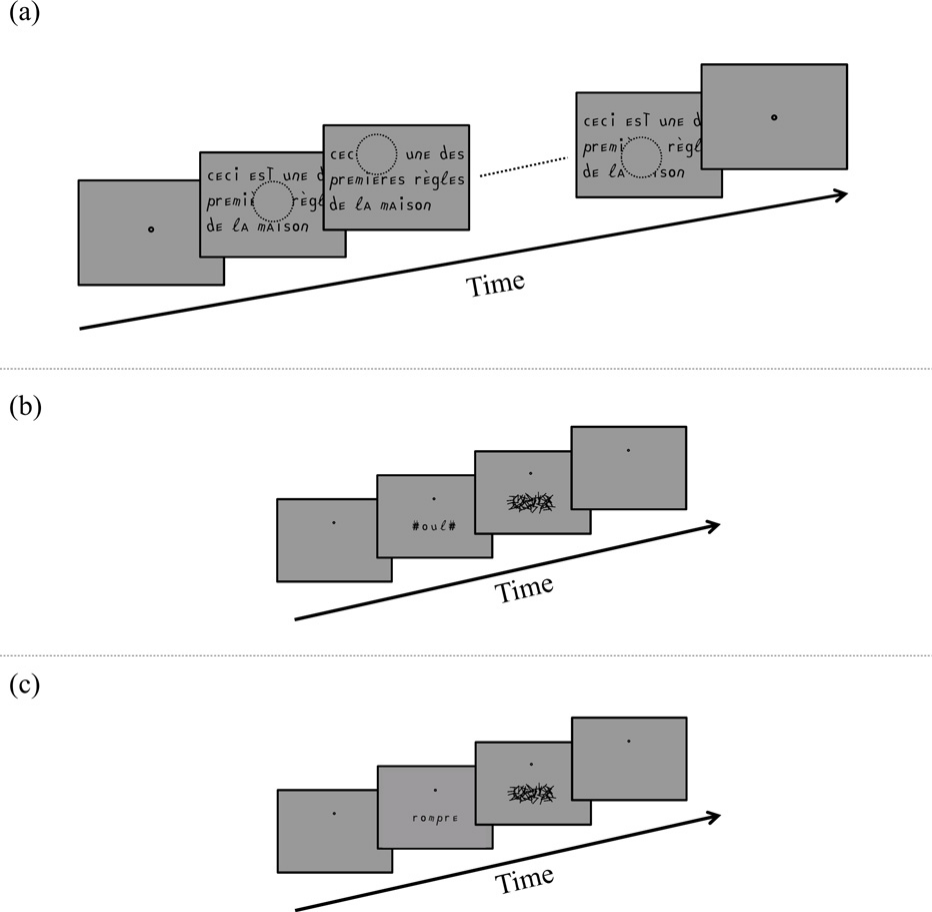
**Description of the experimental protocol for the gaze-contingent reading experiment (Exp1 and 5, plot a), letter recognition experiment (Exp 2 and 6, plot b) and word recognition experiment (Exp 3 and 4, plot c).** In Experiments 1 and 5 (plot a), the subject fixated a dot, pressed a button to display the text (and the gaze-contingent central scotoma centered on the foveal fixation), then made several fixations to identify the words of the sentence while they were reading them aloud (The black circle around the scotoma displayed on the figure was not visible during the experiments and the scotoma was a gray mask with the same luminance as the background). The subject pressed the button when he/she identified the presented words. In Experiments 2, 3, 4 and 6, the subject fixated a dot, pressed a button to display the letter trigram (Experiments 2 or 6, plot b) or the word/pseudoword (Experiments 3 or 4, plot c) in the lower visual field. In Experiments 2, 3 and 6, the stimulus disappeared after the preset presentation time and was replaced by a backward mask (200 ms). In Experiment 4, the stimulus disappeared after the subject pressed one of the two possible buttons.

In Experiment 1, we compared reading performance between the Eido and Courier fonts for six subjects with a gaze-contingent 10° central circular scotoma. Reading speed was measured at seven different print sizes (0.7 to 1.3 logMAR in 0.1 logMAR steps, 0.42 to 1.66 degrees) with the protocol described in Fig 2A: First, subjects had to fixate a dot at the center of the screen. When the observer was ready for the trial, he/she pressed the button of a hand-held joypad. This triggered an eye-tracker offset correction and started the trial: A sentence was immediately displayed centered on the screen. Sentences were displayed similarly to MNREAD principles with 3 or 4 lines of text [15]. Single-line reading speed measurements are usually used to test readers without central vision because the large print-size that needs to be used to make the letters visible does not allow to display a lot of characters or words on a monitor. Each line of text could contain 20 characters (including blank spaces) for any print size. Subjects were asked to read *aloud* the sentence as fast and as accurately as possible. When the subject identified all the words from the sentence (even if he/she did not finish to read aloud the words of the sentence), he/she pressed the button of the joypad and the text was removed from the screen. The difference between the two button presses corresponded to the reading time. At the end of each trial, the experimenter used an interface to report the words that were correctly identified. A built-in program calculated the number of recognized characters for the whole sentences. Reading speed was measured with the following equation following the MNREAD rules [43] and the Carver definition of reading speed [48]:

$$ReadingSpeed\;(words/min)=\frac{nb\_characters\_correctly\_read/6}{Reading\;Time\;(sec)/60}$$

In total, six sentences were presented for each print size and each font. For both fonts, two blocks of 21 trials each were run by each subject with sentence and size randomly selected for each trial. Each subject ran a total of four blocks. The first and fourth blocks were always from the same font (Block 1: Courier font, block 2: Eido font, block 3: Eido font, block 4: Courier font or Block 1: Eido font, block 2: Courier font, block 3: Courier font, block 4: Eido font) to balance the possible effects of learning.

#### Reading performance parameters measurements

**Fig 3:**
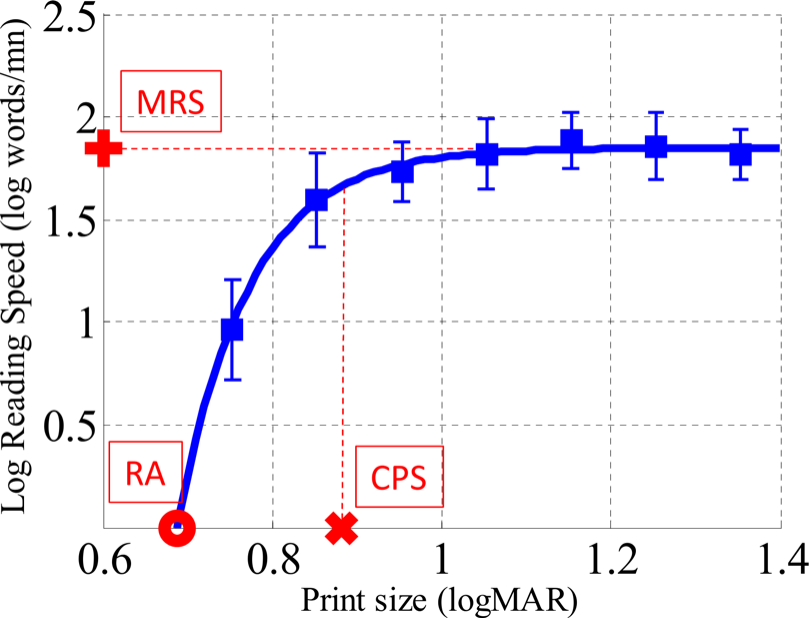
Description of the typical improvement of reading speed in function of letter print-size. First, data are fitted with an exponential function. The maximum reading speed (MRS) corresponds to the asymptote of the curve. The critical print size (CPS) corresponds to the size inducing a reading speed of 90%. The reading acuity (RA) is defined as the intersection between the function curve and the abscissa axis.

Fig 3 shows how reading speed typically improves with print-size only up to the critical print size (CPS), beyond which further increase in print size does not improve reading speed [49,50].The reading speed when letter print size is larger than the CPS is called the Maximum Reading Speed (MRS). Reading Acuity (RA) represents the theoretical minimal print size when reading speed is not null. In Experiment 1, we calculated these three reading performance parameters (CPS, MRS and RA) for each subject. To do so, we fitted experimental data for each subject with the exponential function based on three variables (*φ*1,*φ*2,*φ*3) [51].

$$y=\varphi 1(1-e^{-\varphi 2(x-\varphi 3)})$$

The maximum reading speed was the asymptote of the function (*MRS* = *φ*1). The Critical Print Size was the size corresponding to a reading speed of 90% the MRS ($CPS=\frac{ln(10)}{\varphi 2}+\varphi 3$). The reading acuity was defined as the intersection between the function curve and the abscissa axis (*RA* = *φ*1(1 − *e*^*φ*2*φ*3^)). The largest CPS (max(CPS_Courier_,CPS_Eido_)) obtained in Experiment 1 (average: 0.98, range: 0.73°–1.20°)was the letter print size for our subjects in experiments 2, 3, 4 and 5.

#### (2) Experiments 2 and 6: Peripheral crowded letter recognition

In experiments 2 and 6, we compared peripheral crowded letter recognition between two fonts (Eido and Courier in Experiment 2, and DejaVu and Courier in Experiment 6). Fig 2B schematically describes the temporal course of Experiments 2 and 6: Observers were asked to fixate a dot centered on the screen. No simulated scotoma was used in Experiments 2 and 6. Gaze location was measured to control for steady fixation on the fixation target dot. When the observer was ready for the trial, he/she pressed the button of a hand-held joypad. This triggered an offset correction and started the trial: At 6° eccentricity in the lower visual field, a letter trigram was briefly displayed flanked by two symbols on the left and on the right (each symbol was superposition of the DejaVu and the Courier pound keys). The goal of these symbols was to crowd the extreme trigram letters similarly to the central one). Letter trigrams were displayed in 90% of the trials: Every 10 trials, a single letter was displayed without any flanker to ensure that observers become familiar with the letter shapes of the fonts. A backward mask made of segments (segment stroke-width similar to letter stroke-width) immediately followed the trigram presentation and was displayed for 200 ms. The observer’s answer was stored by the experimenter who gave verbal feedback to the subject in case of letter errors. Each subject ran three sessions (approximately 1h per session), and each session consisted of 4 blocks of 100 trials for each font. The sequence of the blocks was the random succession of block pairs (one block of the Eido/DejaVu font and one block of the Courier font) in a random order. The print size for each observer was obtained in Experiment 1 (average: 0.98°, range: 0.73°–1.20°). The duration for the trigram presentation was determined so that subjects' performance during a pre-test with the Courier font was approximately one letter error per trigram. Average trigram presentation duration was 198 ms (range:110–500 ms).

#### (3) Experiment 3: Peripheral word recognition

In Experiment 3, we compared word recognition performance between the Eido and Courier fonts. The protocol used in Experiment 3 is schematically illustrated in Fig 2C. Similarly to Experiment 2, subjects had to fixate a central dot while a word randomly chosen from our set of words (see previous section) was briefly presented at 6° eccentricity in the lower visual field for each trial, followed by a backward mask made of segments covering the word area. No supplementary flankers were presented on the left and on the right of the word. The observer’s answer was stored by the experimenter who gave verbal feedback to the subject in case of error. Each subject ran two sessions (approximately 1h per session). As in experiments 2, each session was a succession of two pairs of blocks randomly ordered (50 trials per block). For each subject, letter print-size was the same as that used in Experiment 2. Word presentation duration was determined from a preliminary experiment so that subjects reached approximately a 50% word recognition rate. Average word presentation duration was 198 ms (range:50–500 ms).

#### (4) Experiment 4: Peripheral lexical decision

In Experiment 4, we compared reaction times between the Eido and Courier fonts for a lexical decision task in six new normally-sighted subjects. The protocol used in Experiment 4 was similar to the protocol used in Experiment 3 (Fig 2C): For each trial, subjects had to fixate a central dot while a word or pseudo-word randomly chosen from our set of words and pseudowords (see previous section) was briefly presented at 6° eccentricity in the lower visual field. Observers were asked to report as fast as possible if the displayed string of letters was a word or a pseudo-word. This was done by pressing one of two separate buttons of a box. For each trial, the duration time and the observer’s answer were saved. After that, the observer had to press another button and a new word/pseudo-word was displayed two seconds later. Each observer ran two sessions (approximately 45mn per session). As in experiments 2 and 3, each session was a succession of pairs of blocks (one block for the Eido font and one block for the Courier font) randomly ordered (100 trials per block). For each subject, letter print-size was the subject's critical print size (CPS) calculated from a preliminary reading speed experiment similar to Experiment 1. The six subjects' print sizes for this experiment were: 1.08, 1.16, 1.06, 0.94, 1.08 and 1.16 logMAR (1°, 1.20°, 0.96°; 0.73°,1°and 1.20°).

#### (5) Experiment 5: Sentence eye-mediated reading with simulated scotoma

In Experiment 5, six subjects who already ran experiments 1, 2 and 3 had to read sentences with simulated 10° diameter central scotoma. Only one print-size was used for each subject (corresponding to the subject's CPS). Sentences were made of 1 to 8 lines depending on the length of the sentence that was displayed. The experimental protocol for Experiment 5 was similar to Experiment 1 (Fig 2A): A sentence was displayed on the screen as soon as the reader pressed the button a first time. The subject read aloud the sentence by moving his/her eyes (and his/her scotoma), then pressed the button again when he thought he recognized all the words of the sentence. The experimenter reported the words that were correctly identified at the end of each trial, and reading speed was calculated for each trial. Each subject alternatively read blocks of 20 sentences displayed with the Eido font and blocks of 20 sentences displayed with the Courier font. One reading session (approximately 1h) was run for each subject, which corresponded to a total of 120 sentences in average per subject.

### Statistical Analysis

Statistical analyses were performed using the language and environment R [52]. We investigated the effects of font type and other factors on dependent variables by using linear mixed-effects models (function lme from the nlme package [53,54]) and generalized linear mixed-effects models (function glmer of the lme4 package). Continuous independent variables were centered on their mean. In the case of the font variable, Courier was always the reference level. P-values were based on conditional t-tests (see [53] page 90).

## Results

### Experiment 1: Sentence reading with simulated scotoma

**Fig 4:**
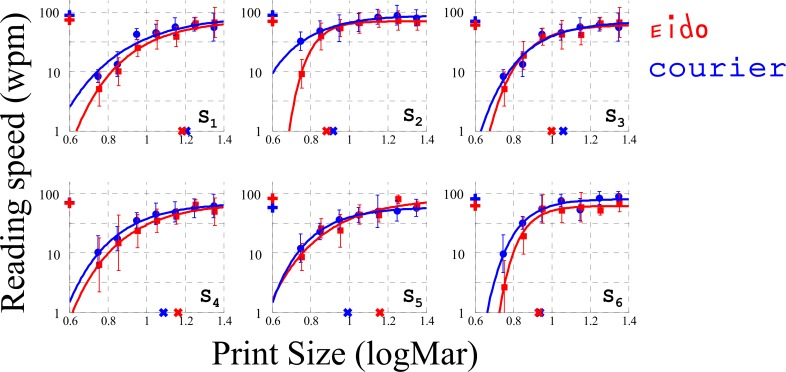
Sentence reading with simulated scotoma (Experiment 1). Effect of print-size (x-height, logMar) on reading speed (words per minute) for each of the six subjects. For both fonts, the '+' on the ordinate axis represents the maximum reading speeds (MRS) and the 'x' on the abscissa axis represents the Critical Print Size.

**Fig 5:**
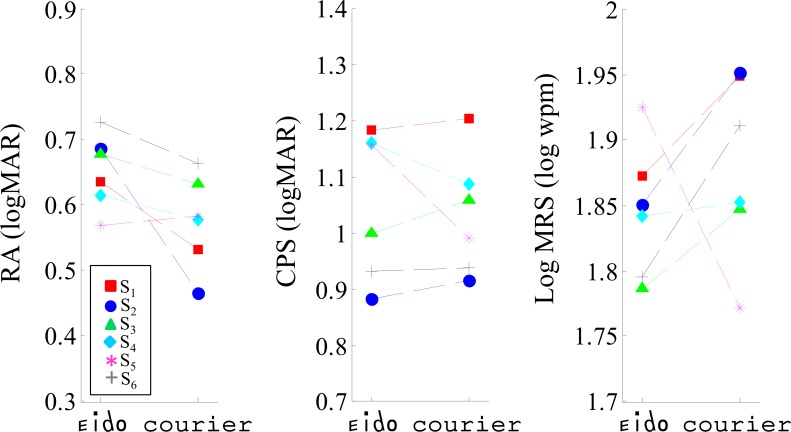
Sentence reading with simulated scotoma (Experiment 1). Effect of font type on the three extracted reading parameters: reading acuity (RA), Critical Print Size (CPS) and log Maximum Reading Speed (logMRS).

The goal of Experiment 1 was (1) to test whether the relationship between reading speed and letter print size was different between the Eido and the Courier fonts and (2) to define a print-size for the following experiments. Fig 4 shows the effect of print size on sentence reading speed for the Eido and Courier fonts for the different subjects. The exponential function described in the methods section was used to fit the data for each subject. Based on these curves, we extracted the three main characteristics of reading performance for the six subjects and for both fonts: the Critical Print Size (CPS), log Maximum Reading Speed (logMRS) and Reading Acuity (RA). These characteristics are plotted in Fig 5 for both fonts. Statistical analysis was performed using linear mixed effects modeling with random intercept and random slope to assess the effect of font type on the three reading parameters (fixed effects: Font type, random effects: Subject factor). Results for the three models are indicated in Tables 1–3. They do not show any significant effect of the font on log Maximum Reading Speed (p = 0.42), Critical Print Size (p = 0.58) and Reading Acuity (p = 0.07). The marginal effect observed for reading acuity suggests that the EIDO font could alter reading performance for low print-size, probably because of the low complexity of its letters decreasing its visual acuity [55]. The main result is therefore that the relationship between reading speed and print size is similar between the Eido and the Courier font. This absence of an effect is contrary to our expectations and is interpreted in the discussion based on the results of the four experiments. The print sizes used in experiments 2–6 were determined based on the CPS values measured in Experiment 1. Print-size used for these experiments was max(CPS_Courier_,CPS_Eido_).

**Table 1:** Fixed effects results of the linear mixed-effects model in Experiment 1 (Dependent Variable is Maximum Reading Speed).

	Estimate	Std.Error	DF	t-value	p-value
(Intercept)	1.85	0.02	5	88.50	0.00001
Font	0.04	0.04	5	0.87	0.42

Results show the effect of font type (Reference level: Courier) on Maximum Reading Speed (MRS).

**Table 2:** Fixed effects results of the linear mixed-effects model in Experiment 1 (Dependent Variable is Critical Print Size).

	Estimate	Std.Error	DF	t-value	p-value
(Intercept)	1.05	0.05	5	19.55	0.00001
Font	-0.02	0.03	5	-0.59	0.58

Results show the effect of font type (Reference: Courier font) on Critical Print Size (CPS).

**Table 3:** Fixed effects results of the linear mixed-effects model in Experiment 1 (Dependent Variable is Reading Acuity).

	Estimate	Std.Error	DF	t-value	p-value
(Intercept)	0.65	0.02	5	28.31	0.00001
Font	-0.08	0.03	5	-2.31	0.07

Results show the effect of font type (Reference: Courier font) on Reading Acuity (RA).

### Experiment 2: Crowded letter recognition

**Fig 6:**
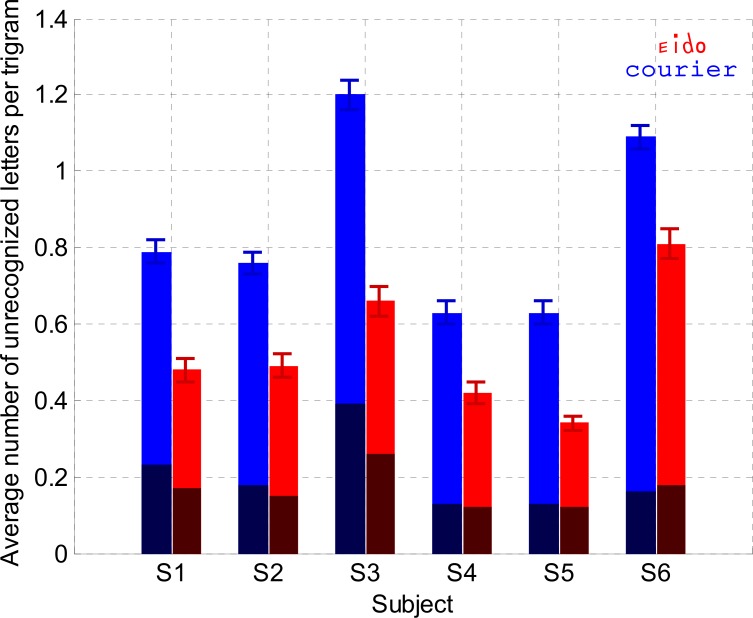
Crowded letter recognition (Experiment 2). Average number of unrecognized letters per trigram for each subject and for both fonts (red color: Eido font, blue color: Courier font). Standard deviations were obtained by bootstrapping for the distribution of errors per trigram (1,000 simulation trials). The dark part of the bars corresponds to letter errors due to letter mislocations.

The goal of Experiment 2 was to test if the Eido font could improve letter legibility under crowding as expected from its development. To test this hypothesis, we determined the effect of font type (Eido vs. Courier) on crowded letter recognition performance by calculating the number of unrecognized letters and the number of mislocalized letters (letters that were correctly recognized but incorrectly localized) per trigram. For instance, if the trigram ‘bon’ was presented and the subject named the trigram ‘beo’, then one letter was correctly recognized (the letter 'b') and one letter was mislocalized (the letter ‘o’). Fig 6 shows for each subject the average number of letters incorrectly recognized per trigram for the Eido and Courier fonts. Fig 6 also indicates the average number of letters mislocalized per trigram. Linear mixed effects modeling was performed to determine the difference between both fonts in (1) letter recognition and (2) letter mislocation errors. Random effect was the subject factor. Fixed effects were the font type factor, the block number factor (centered on the average block number) and their interaction. This interaction between the block number and the font type aimed at testing the potential larger learning effect with the Eido font compared to the Courier font (given the lack of experience of the subjects with this font). In the first analysis, the number of errors was the dependent factor. Results in Table 4 show that both the block number and the font type were significant predictors of letter crowded recognition performance (p<10^−5^ for each factor). On average, there was a significant decrease of 0.03 letter error per block, with no interaction between the block number factor and the font factor (p = 0.67). The most important result of the analysis was that crowded letter errors were significantly lower (-29.63% on average) with the Eido font compared to the Courier font (1.08 errors per trigram on average for the Courier font compared to 0.76 errors per trigram for the average block number for each subject). A second mixed effects model was performed to analyse the effect of block number and font type on the number of letter mislocation errors. Results in Table 5 show that there was a significant effect of the block number on the number of mislocalized letters per trial (p<10^−4^) with an average decrease of 0.008 letter mislocations per block. There was no significant correlation between the block number and the font factors (p = 0.87). There was a marginal effect of the font type on mislocation errors (p = 0.07; on average, 0.26 mislocalized letters per trigram for the Courier font vs. 0.22 mislocalized letters per trigram for the Eido font for the average block number). To summarize, we found a significant decrease of letter recognition errors with the Eido font compared to the Courier font (-30% on average), with a marginal difference in the number of mislocalized letters between both fonts.

**Table 4:** Fixed effects results of the linear mixed-effects model for Experiment 2 (Dependent Variable is the number of letter errors).

	Estimate	Std.Error	DF	t-value	p-value
(Intercept)	1.08	0.14	8530	7.62	0.00001
#block	-0.03	0.008	8530	-3.54	0.0004
Font	-0.32	0.04	8530	-8.73	0.00001
#block*Font	0.002	0.004	8530	0.42	0.67

Results show the effects of the block number, the effect of the font type (Reference: Courier font) and their interaction on the number of letter errors.

**Table 5:** Fixed effects results of the linear mixed-effects model for Experiment 2 (Dependent Variable is the number of mislocalized letters).

	Estimate	Std.Error	DF	t-value	p-value
(Intercept)	0.26	0.04	8530	5.98	0.00001
#block	-0.008	0.002	8530	-3.42	0.0006
Font	-0.04	0.02	8530	-1.82	0.07
#block*Font	0.0004	0.002	8530	0.16	0.87

Results show the effect of the block number, the effect of the font type (Reference: Courier font) and their interaction on the number of mislocalized letters.

### Experiment 3: Single Word recognition

**Fig 7:**
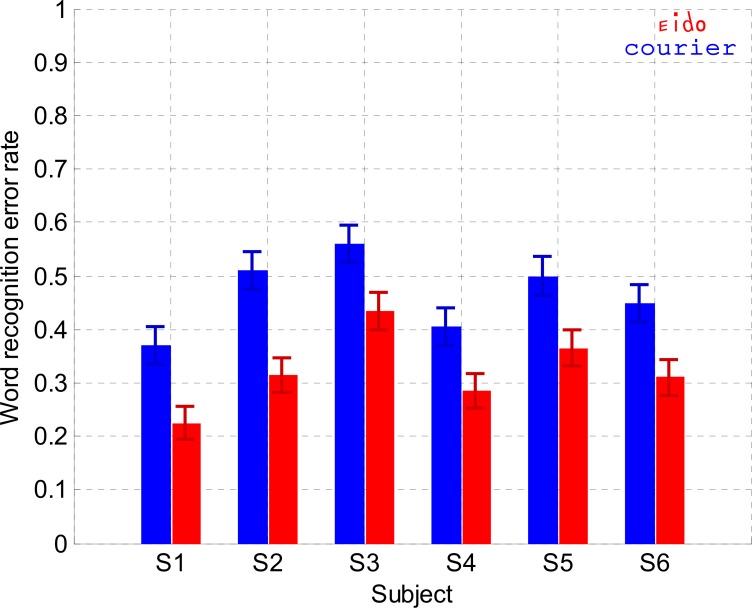
Word recognition (Experiment 3). Word recognition error rate for each subject, for the Eido font (red) and the Courier font (blue).

The goal of Experiment 3 was to test if the Eido font could also increase word recognition performance compared to the Courier font. Fig 7 shows the word recognition error rates for the Eido and Courier fonts. A word was incorrectly recognized if at least one letter was incorrectly identified. A generalized linear mixed model for binary responses was performed to analyse the word recognition performance. The random effect was the subject factor and the fixed factors were the font type, the word length (centered on the average word length), the word frequency (centered on the average word frequency) and the block number factors (centered on the average block number). The dependent variable was the word recognition error variable (0 or 1). Results of the analysis in Table 6 indicate that the block number had no significant effect on word recognition error rate (p = 0.07). On the contrary, word length and word frequency were significant predictors of word recognition error rate (p<10^−5^ and p<10^−4^). Word recognition error rate increased by 5.25% on average for each additional character when all other factors were at their reference value (Courier font, average block and average word frequency). Word recognition error rate decreased by 7.92% on average when there was a word frequency increase of one log occurrence per million when all other factors were at their reference value (Courier font, average block and average word length). In this analysis, the most important result is the significant effect of the font type: Word recognition error rate decreased on average from 0.46 for the Courier font to 0.32 for the Eido font when all other factors were at their reference value: average block, average word frequency and average word length. To summarize, when compared with the Courier font, the Eido font significantly reduced the errors made during peripheral word recognition task by 32.03% on average across subjects.

**Table 6:** Fixed effects results of the generalized linear mixed-effects model for Experiment 3 (Dependent Variable is the word recognition error rate).

	Estimate	Std.Error	z-value	p-value
(Intercept)	-0.14	0.12	-1.17	0.24
#block	-0.04	0.02	-1.82	0.07
Font	-0.63	0.09	-7.21	0.00001
Word length	0.21	0.03	7.18	0.00001
Word frequency	-0.0007	0.0003	-2.03	0.04

Results show the effect of block number, font type (Reference: Courier font), word length and word frequency on word recognition error rate.

#### Linking word recognition performance and letter recognition performance

**Fig 8:**
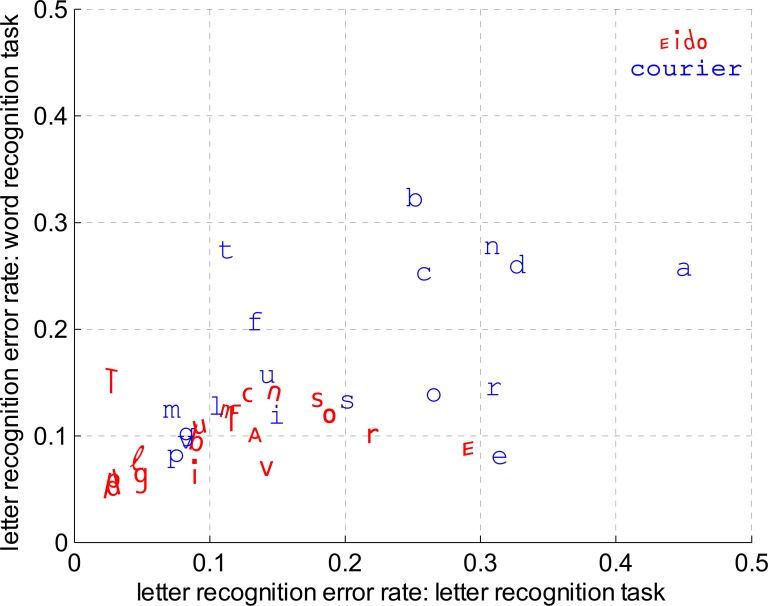
Letter recognition error rate (word recognition task) vs. Letter recognition error rate (letter recognition task). Letter recognition error rate during the word recognition task (Experiment 3) as a function of letter recognition error rate during the letter recognition task (Experiment 2), averaged across subjects for each letter and both fonts. For instance, the position of the letter 'n' for the Courier font shows that the letter recognition error rate was about 0.31 for the letter recognition task and 0.27 for the word recognition task when the Courier font was used.

In this analysis, we investigated the link between letter recognition error rate occurring during the letter recognition task (Experiment 2) and letter recognition error rate occurring during the word recognition task (Experiment 3). Fig 8 shows for each letter in both fonts and both tasks the recognition errors averaged across subjects. We performed a statistical analysis based on a generalized linear mixed-effects model to test if we could predict the letter recognition error rate for each individual letter during the word recognition task, based on the letter recognition error rate for each individual letter during the letter recognition task. Random effects were the subject factor and the letter factor, and the fixed effects were the font factor and the arcsin transform of the proportion of errors for each letter during the letter recognition task. The dependent variable was the proportion of errors for each letter during the word recognition task. Results of the analysis are shown in Table 7. They indicate that the proportion of errors for each individual letter during the letter recognition task and the font type are both significant predictors of proportions of errors for each individual letter during the word recognition task.

**Table 7:** Fixed effects results of the generalized linear mixed-effects model to predict the proportion of recognition errors for each letter during the word recognition task.

	Estimate	Std.Error	z-value	p-value
(Intercept)	-1.41	0.13	-10.72	0.00001
#block	0.43	0.007	58.36	0.00001
Font	-0.36	0.004	-80.35	0.00001

Results show the effect of the proportion of recognition errors for each letter during the letter recognition task and the effect of the font type (Reference: Courier font) on the proportion of errors for each letter during the word recognition task.

### Experiment 4: Lexical decision

**Fig 9:**
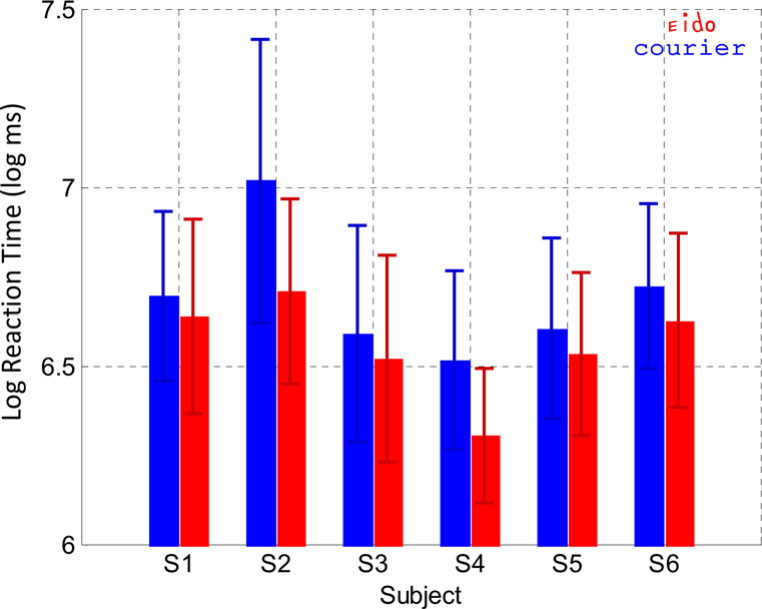
Word lexical decision (Experiment 4). Average and standard deviation of the log lexical decision time for each subject and for both fonts (red color: Eido font, blue color: Courier font).

The goal of Experiment 4 was to test if the Eido font could also accelerate peripheral word processing compared to the Courier font, in addition to increase peripheral word recognition rate as shown in Experiment 3. Fig 9 shows the log lexical decision times for the Eido and Courier fonts for each subject. Subjects were very good in this task with correct lexical decision rates superior to 86% for each subject and each font (average: 94% for the Eido font and 92% for the Courier font). Linear mixed effects modeling was performed to determine the effects of font type on lexical decision time. Only word trials were considered for analysis. Outliers (response times slower than three standard deviations above the subject's average) were removed for each subject independently (2–5 trials per subject). The random effect was the subject factor and the fixed factors were the font type, the word length (centered on the average word length), the word frequency (centered on the average word frequency), the number of orthographic neighbors (centered on the average number of orthographic neighbor) and the block number factors (centered on the average block number). The dependent variable was the natural logarithm of lexical decision time (in log ms). Results of the analysis in Table 8 indicate that the block number had no significant effect on word lexical decision time (p = 0.32). Among the orthographic and linguistic variables, the number of orthographic neighbours is not a significant predictor of lexical decision time (p = 0.93). On the contrary, word length and word frequency were significant predictors (p<10^−5^). Lexical decision time increased by 0.07 log ms on average for each additional character when all other factors were at their reference value (Courier font, average block, average word frequency and average number of orthographic neighbors). Lexical decision time decreased by 0.04 log ms on average when there was a word frequency increase of one log occurrence per million when all other factors were at their reference value (Courier font, average block, average word length and average number of orthographic neighbors). In this analysis, the most important result is the significant effect of the font type: Lexical decision time decreased on average from 6.73 log ms (837 ms) for the Courier font to 6.61 log ms (741 ms) for the Eido font when all other factors were at their reference values (average block, average word frequency, average word length and average number of orthographic neighbors). To summarize, when compared with the Courier font, the Eido font significantly reduced the lexical decision time by 96 ms on average across subjects.

**Table 8:** Fixed effects results of the linear mixed-effects model for Experiment 4 (Dependent Variable is the log lexical decision reaction time).

	Estimate	Std.Error	DF	t-value	p-value
(Intercept)	6.73	0.09	1801	74.90	0.00001
#block	-0.01	0.01	1801	-1.00	0.32
Font	-0.12	0.01	1801	-9.50	0.00001
Word length	0.07	0.007	1801	9.53	0.00001
Word frequency	-0.04	0.006	1801	-6.49	0.00001
Word_nb_ortho._neighbors	0.0003	0.004	1801	0.05	0.93

Results show the effects of block number, font type (Reference: Courier font), word length, word frequency and number of orthographic neighbors on lexical decision reaction time.

### Experiment 5: Sentence eye-mediated reading with simulated scotoma

**Fig 10:**
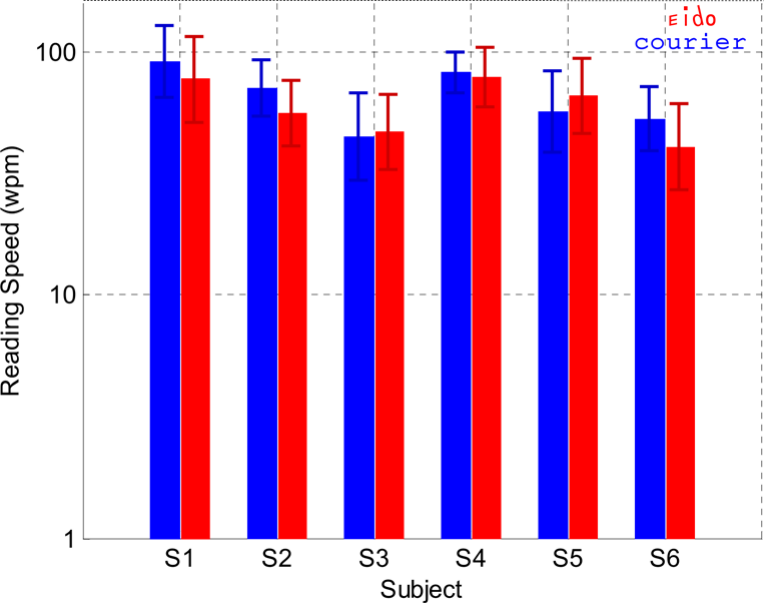
Sentence reading with simulated scotoma (Experiment 5). Average and standard deviation log reading speed for each subject and for both fonts (red color: Eido font, blue color: Courier font).

The goal of Experiment 5 was to test if the Eido font could have an effect on reading speed after subjects had had several hours of training from previous experiments in sentence reading (Experiment 1), letter recognition (Experiment 2) and word recognition (Experiment 3). These experiments represented several thousand of letters and words to be recognized. So, it is possible that an effect of the Eido font on reading speed could be visible after this training opportunity compared to Experiment 1 when subjects were not familiar with the Eido font. Fig 10 shows the reading speeds for the different subjects who read sentences from a French novel with the Eido font and with the Courier font. Linear mixed effects modeling was performed on data to find a possible effect of font type on reading speed. The random effect was the subject factor. The fixed factors were the font type, the block number (centered on the average block number) and their interaction. This interaction between the block number and the font type aimed at testing the possible difference in learning between both fonts. The dependent variable was the logarithm of reading speed (in log words/mn). Results of the analysis in Table 9 indicate that the block number had a significant effect on reading speed (p = 0.04). On the contrary, the font type was not a significant predictor (p = 0.13). No interaction between the font type and the block number was clearly significant (p = 0.95). These results confirm the results of Experiment 1: the Eido font did not increase reading speed performance, even after the large letter recognition and word recognition training that occurred in Experiment 1, 2 and 3 for the 6 subjects.

**Table 9:** Fixed effects results of the linear mixed-effects model for Experiment 5 (Dependent Variable is the log reading speed).

	Estimate	Std.Error	DF	t-value	p-value
(Intercept)	1.73	0.06	625	29.38	0.00001
#block	0.037	0.02	625	1.52	0.13
Font	0.01	0.005	625	2.01	0.04
#block*Font	0.0003	0.005	625	0.07	0.95

Results show the effect of the block number, the effect of the font type (Reference: Courier font) and their interaction on the log reading speed.

### Experiment 6: Crowded letter recognition (Control experiment)

**Fig 11:**
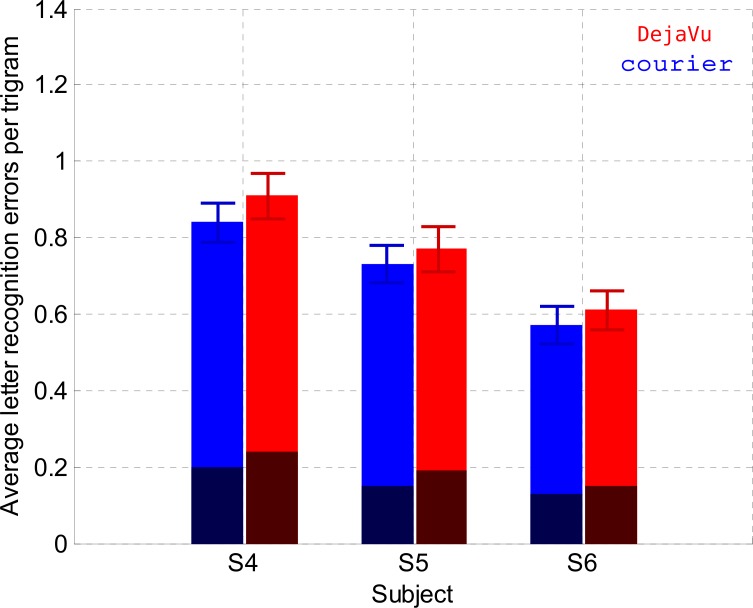
Letter recognition (Experiment 6). Average number of unrecognized letters per trigram for each subject and for both fonts (red color: DejaVu font, blue color: Courier font). Standard deviations were obtained by bootstrapping for the distribution of errors per trigram (1,000 simulation trials). The dark part of the bars corresponds to letter errors due to letter mislocations.

We designed our previous experiments so that center-to-center spacing was identical between the Courier and Eido fonts (Fig 1). However, the reduced complexity of the Eido font induced a larger edge-to-edge spacing between Eido letters. The goal of Experiment 6 was to ensure that the effect of font type that we found in crowded letter recognition and word recognition experiments was not due to a reduction in edge-to-edge spacing. For this purpose, we ran a new experiment to compare the Courier font with the DejaVu font, a font with letter templates similar to Courier font, but an edge-to-edge spacing very similar to the Eido font (0.27*x-height). Fig 11 shows the results for the three subjects who ran this experiment. There is clearly no advantage in using the DejaVu rather than the Courier font to recognize words. A linear mixed effects model was used to determine the effect of font type on crowded letter recognition. The random effect was the subject factor, and the fixed effects were the font and the block number (centered on the average block number) factors. The dependent variable was the number of letters errors within a trigram. Results of the analysis are shown in Table 10. They indicate that the number of letter errors for the DejaVu font is not significantly different compared to the number of letter errors for the Courier font (p = 0.73). Results of Experiment 6 suggest that the differences in letter and word recognition performance between the Eido and Courier fonts are not due to a difference in edge-to-edge spacing between both fonts.

**Table 10:** Fixed effects results of the linear mixed-effects model for Experiment 6 (Dependent Variable is the number of letter errors).

	Estimate	Std.Error	DF	t-value	p-value
(Intercept)	0.73	0.09	1614	8.57	0.00001
#block	-0.09	0.03	1614	-2.85	0.004
Font	0.02	0.04	1614	0.35	0.72

Results show the effect of the block number and the effect of the font type (Reference: Courier font) on the number of letter errors.

## Discussion

In this study, we investigated a new font, named the Eido font, that we specifically designed to increase the legibility of letters presented in peripheral vision. The Eido letters have been designed based on three main principles: (1) Reducing physical similarities between letters, (2) Reducing letter visual complexity [18] and (3) Designing letter shapes relatively familiar to observers. Our purpose was to test if the Eido font could increase peripheral letter recognition performance despite crowding, and in consequence peripheral reading speed [4,53] compared to the Courier font, a font with traditional letter shapes.

Results from Experiment 1 (Fig 3) and Experiment 5 (Fig 10) showed that the Eido font was not able to significantly increase peripheral sentence reading speed when subjects read with a simulated 10° diameter central scotoma. However, results from experiments 2 and 3 showed that the Eido font was able to significantly increase peripheral crowded letter recognition rate and word recognition rate when letters and words were briefly presented at 6° eccentricity, while observers fixated a dot. With the Eido font, peripheral letter recognition errors decreased by 30% on average across subjects (Fig 6) and peripheral word recognition error rate decreased by 32% averaged across subjects (Fig 7). Results from Experiment 4 also showed that lexical decision times could be decreased by 96 ms on average across subjects (Fig 9).

The improvement in crowded letter recognition performance validates the design of the Eido font as the legibility of letters in crowded conditions has been increased compared to the Courier font. Fig 8 shows that, on average, frequent confusions (confusion rate superior to 20%) occurred for nine letters with the Courier font (letters 'b', 'c', 'o', 'n', 'r', 'a' and 'e') and only for two letters with the Eido font (letters 'r' and 'e'). Interestingly, our new font significantly reduced the number of letter confusions, but the number of letter mislocations, which represents a large part of usual letter crowding errors [19,20], did not significantly change compared to the Courier font, suggesting an interesting dissociation between letter confusion and letter mislocation errors during crowding. Based on this result, the amount of letter mislocation errors could be considered as an independent source of errors caused by source confusion at a letter symbol level [19,20,54], rather than at a letter feature level [18,25].

The Eido font also significantly reduced word recognition errors compared to the Courier font. This result can appear surprising for at least two reasons: (1) Word shape information may be useful in reading [55] and is strongly modified by the Eido font compared to traditional fonts because of the presence of oblique lines within the words. Especially, ascender and descender oblique lines may deteriorate the localization of ascender and descender letters within the words. (2) It has been shown that too much distinctiveness between letters within a font may have a negative impact on letter and word recognition (font tuning) [56]. It has also been shown that the presence of mixed-case letters among words (as it is the case in a lot of Eido words because of the letters 'a', 'e' and 't') can reduce foveal word recognition and reading speed [57–59]. However, the results for the word recognition experiments (experiments 3 and 4) suggest that the deleterious effects caused by the unfamiliar Eido word shapes and the distinctive Eido letters are not sufficient to counterbalance the positive effect concerning the large improvement in peripheral letter recognition performance. Confirming this hypothesis, Fig 8 suggests that letters that were frequently confused during the crowded letter recognition task were also frequently confused during the word recognition task. This result is a direct support of theories that describe letter recognition as the necessary step preceding word recognition [5]. Experiment 6 confirms that this result is specifically due to the Eido letter shapes and not the large letter edge-to-edge spacing difference between the Eido and Courier font.

Why did the Eido font significantly improve letter recognition rate, word recognition rate and speed up word recognition processing for each of our subjects, but did not improve eye-mediated sentence reading performance? Theoretically, the visual information extracted at each fixation should be increased with the Eido font, which could increase the size of the visual and perceptual span [60,61], consequently increasing the eye-mediated reading rate [62,63]. One possible explanation could be that the perceptual improvement in letter and word recognition may not be large enough to overcome the oculo-motor difficulties of subjects without central vision. Indeed, in addition to perceptual difficulties to extract visual information at each fixation, readers with central field loss have difficulties to position and stabilize their fixations [64,65]. These difficulties in oculomotor control increase the number of fixations made by the reader to recognize a single word, inducing supplementary trans-saccadic integration difficulty [66,67]. Thus, the relative part of the perceptual gain due to the Eido font is probably strongly reduced in presence of all these new factors limiting the recognition of words when the eyes can freely move. Suggesting a similar explanation, a previous study showed the dramatic difference that can exist for the effects of a visual aid (large interline spacing) on peripheral reading speed between static reading (without eye movements, +178% increase) and dynamic reading (with eye movements, +26% increase) [15]. Altogether, these results suggest that letter identification may not be the main limiting factor in sentence reading with central field loss. The role of attentional, oculo-motor and trans-saccadic integration limitations could be critical to explain the dramatic reduction in eye-mediated reading performance for readers without central vision.

To conclude, future experiments will have to study the effect of longer training period with the Eido font on letter recognition, word recognition and eye-mediated reading performance. Even if our subjects were already familiar with the Eido font when they ran Experiment 5 after experiments 1, 2 and 3, learning new letter templates usually necessitates a large number of trials [68]. Because one consequence of this learning could be the reduction of the negative font tuning effect [69], a longer training could probably improve peripheral as well as foveal reading performance given the influence of peripheral letter recognition performance on reading speed [64]. However, in these dynamic foveal reading situations, it has been suggested that parafoveal crowding could have little effect on reading performance [70–72]. From a theorical perspective, our results concerning the different effects of the Eido font on letter/word recognition and on eye-mediated reading performance are interesting to understand the different weights of perceptual and oculomotor factors on peripheral reading performance. From a clinical perspective, the Eido font could be helpful for low vision patients when used to display single letters and words on specific product labels or in low vision centers. The Eido font will finally need to be tested with low vision patients to confirm its promising interest and to completely understand its lack of effect in eye-mediated reading.

## Supporting Information

S1 Appendix Formulas used to realize the different mixed-effect models shown in Tables 1–10. (DOCX)
