# Supplementary material for: A New Font, Specifically Designed for Peripheral Vision, Improves Peripheral Letter and Word Recognition, but Not Eye-Mediated Reading Performance
Source: PLoS One. 2016 Apr 13;11(4):e0152506. doi: 10.1371/journal.pone.0152506 (PMC4830533; doi:10.1371/journal.pone.0152506)
Supplement: S1 Appendix — (DOCX) [file pone.0152506.s001.docx]

APPENDIX

Formulas for Table 1-3

**model1 = lme(MRS ~ Font, data = data_all,**

**random = ~ 1 + Font|Subject,**

**)**

**model2 = lme(CPS ~ Font, data = data_all,**

**random = ~ 1 + Font|Subject,**

**)**

**model3 = lme(RA ~ Font, data = data_all,**

**random = ~ 1 + Font|Subject,**

**)**

Formula for Table 4

**model = lme(letters_not_recognized ~ n_block * Font, data = data_all,**

**random = ~ 1 + n_block| Subject,**

**)**

Formula for Table 5

**model = lme(letters_mislocalized ~ n_block * Font, data = data_all,**

**random = ~ 1 + n_block| Subject,**

**)**

Formula for Table 6

**model = glmer(word_correctly_recognized ~ n_block + Font + word_length + word_frequency + (n_block|Subject), data_all, family='binomial')**

Formula for Table 7

**model = glmer(percent_word ~ percent_letter * Font + (1|letter.id) + (1|Subject),binomial,data=data,weights=n_letters)**

Formula for Table 8

**model = lme(Log_lexical_decision_time ~ Font + length + frequency + n_orthographic_neighbors + n_block, data = data_all,**

**random = ~ 1 + n_block| Subject,**

**)**

Formula for Table 9

**model = lme(logRS ~ n_block * Font, data = data_all,**

**random = ~ 1 + n_block| Subject,**

**)**

Formula for Table 10

**model = lme(letters_correctly_recognized ~ n_block + Font, data = data_all,**

**random = ~ 1 + n_block| Subject,**

**)**
